# GLP-1 based therapy of obesity and type 2 diabetes

**DOI:** 10.1007/s13340-026-00946-x

**Published:** 2026-11-10

**Authors:** Jens Juul Holst

**Affiliations:** The NovoNordisk Foundation Center for Basic Metabolic Research and Department of Biomedical Sciences, The Panum Institute, University of Copenhagen, Blegdamsvej 3, Copenhagen N, DK 2200 Denmark

**Keywords:** Glucagon-like peptide-1, Proglucagon, Glicentin, Ileal brake, Semaglutide, Tirzepatide

## Abstract

GLP-1 is 30-amino acid peptide that is secreted from the intestine in response to food intake. The release mechanism involves luminal exposure to as well as absorption and metabolism of the major nutrients. It functions mainly as a part of the ileal brake mechanism whereby nutrient entry in distal segments of the small intestine inhibit gastrointestinal secretion and motility as well as appetite and food intake, signalling nutritional abundance. It also functions as a second line incretin hormone. It acts on the brain via sensory vagal afferents, but also interacts with exposed neurons expressing the GLP-1 receptor in the circumventricular organs, in turn activating brain nuclei involved in appetite regulation and reward. High doses of GLP-1 cause nausea and vomiting, but slow dosing titration mitigates this. GLP-1 Receptor Agonists owe their actions to these physiological mechanisms and therefore represent powerful antidiabetic agents (A1c levels below 5.7%) and cause major weight loss (up to 20%), but also appear to reduce the systemic low-grade inflammation of obesity and type 2 diabetes and to exert beneficial action on the blood vessels, providing the GLP-1RAs with preventive effects on cardiovascular, hepatic, renal and central nervous system complications. Orally active GLP-1rAs have also been developed. It is emphasized that GLP-1RA therapy should be accompanied by lifestyle changes for efficacy and to prevent side effects of the weight loss. In addition, any prescription of a GLP-1RA should include a plan to mitigate adverse effects of treatment termination. Nevertheless, GLP-1RAs represent a major breakthrough in the therapy of metabolic disease and are now included among the essential medicines of the WHO.

## Physiology of GLP-1

### Localization and secretion; the proglucagon products

GLP-1 is a 30-amino acid peptide product of expression of the proglucagon gene in the endocrine L-cells of the intestinal epithelial L-cells [1]. The L-cells are of the open type, meaning that they possess cytoplasmatic processes that extend into the lumen of the gut [2]. The processes are equipped with microvilli which express proteins involved in digestion and absorption, including glucose transporters (SGLT-1) and amino acid transporters [3]. They may also express certain lipid receptors, for instance GLR 119, a target of diacylglycerols [4]. This suggests that the L-cells can sense the presence of certain nutrients in the gut lumen and respond to their presence with secretion from the basolateral membranes. The L-cells are found throughout the intestinal system and with increasing density distally (even in the distal colon), but the normal meal response is probably elicited by nutrients presented to the proximal small intestine [5]. The primary translation product of the proglucagon gene, a 160 amino acid peptide, is also formed in the pancreatic alpha cells but is processed differentially in the two tissues giving rise to the following peptide products: In the *pancreas*, a fragment of proglucagon (PG) corresponding to residues no 1–30 (GRPP, glicentin-related pancreatic polypeptide) ; glucagon (residues no 33–61 of PG) and the so-called major proglucagon fragment (MPGF, PG 72–158/160), and in the *gut* to glicentin (PG 1–69), GLP-1 (PG 78–107) and GLP-2 (PG 126–158) [6]. The differential cleavage is due to expression of different prohormone convertases in the two tissues, namely PC2 in the pancreas and PC 1/3 in the gut [1].

Among the pancreatic products of proglucagon, the only one exhibiting significant biological activity is glucagon itself; neither the GRPP nor the MPGF have been reported to exert significant activities. Among the intestinal products, glicentin is probably also inactive, consistent with the inactivation of most peptides of this family by N-terminal extensions (in this case of the glucagon moiety of glicentin). Glicentin is, however cleaved to produce a 37 amino acid peptide, oxyntomodulin, corresponding to PG 33–69 (and GPRR) [7]. Oxyntomodulin interacts with the glucagon receptor although less potently (1: 100) than glucagon, but unexpectedly also interacts with the GLP-1 receptor again with weaker potency. Nevertheless, the dual activity has inspired development of glucagon-GLP-1 co-agonists [8], which have significant metabolic effects and distinguish themselves from GLP-1 mono-agonist by their specific actions on liver metabolism (a glucagon action). Because of glucagon’s effects on hepatic lipid metabolism, they appear highly effective in the treatment of MASLD, steatosis of the liver [9] (see below). The three remaining gut products are the glucagon-like peptides 1 (PG 78–107) and 2 (PG126-158), and the second intervening peptide (IP2, PG 111–123), the latter of which is poorly explored. GLP-2 mainly acts as a growth factor for the intestinal mucosa, but will not be dealt with further here except to note, that a dual GLP-1/GLP-2 agonist has been developed and is investigated for metabolic effects [10]. It should be noted here that the L-cells, apart from the proglucagon derived peptides, also produce and secrete peptide YY (PPY ), a 36 amino acid peptide, in parallel with GLP-1, at least from the distal L-cells [11], and this peptide also has significant effects on food intake [12]. So far it has been difficult to harness its effect on food intake into an appropriate pharmaceutical form, but studies are still ongoing and are obviously highly relevant, since the physiological effects of L-cell secretion are due to the release of all its peptide products [13]. A time line for the discovery of GLP-1 and the development of the GLP-1 receptor agonists (GLP-1RAs) is given in Table 1.

**Table 1:** Important steps in the development of the GLP-1 Receptor agonists

1983: Cloning of a mammalian proglucagon (hamster): identification of two glucagon-like sequences in the cDNA [14]
1986: Demonstration of proglucagon in the pancreas and the gut; the differential cleavage and secretion: glucagon from the pancreas, GLP-1 and GLP-2 from the gut ADDIN EN.CITE [15, 16]
1986-87 : The sequence of natural GLP-1 (GLP-1 7-37/36amide) and its insulinotropic activity ADDIN EN.CITE [17, 18]
1987: Incretin function of GLP-1 in man [19]
1988: inhibition of glucagon secretion by GLP-1 [20]
1993: Normalization of plasma glucose with GLP-1 infusion in T2DM ADDIN EN.CITE [21]
1993: Inhibition of gastric emptying and gastro-pancreatic secretion in man ADDIN EN.CITE [22]
1992: Cloning of the GLP-1 receptor by Bernard Thorens ADDIN EN.CITE [23].
1992: Activation of GLP-1 receptors by exendin 4 from the Gila monster saliva ADDIN EN.CITE [24]
1995: DPP-4 found to be responsible for the short half-life (2 min) of GLP-1 in the circulation; DPP-4 inhibitors for diabetes therapy ADDIN EN.CITE [25, 26]
1998: Inhibition of appetite and food intake by GLP-1 in humans ADDIN EN.CITE [27].
2002: Demonstration of the effects of chronic (6 weeks) treatment of T2DM with subcutaneously infused GLP-1: positive effects on HbA1c, body weight; beta cell function; insulin resistance ADDIN EN.CITE [28].
2005: Approval of synthetic exendin 4 (exenatide) for diabetes therapy [29]
2006: Approval of the first DPP-4 inhibitor (sitagliptin) for diabetes therapy ADDIN EN.CITE [30]
2009: Approval of liraglutide, the first long-acting (acylated) GLP-1 receptor agonist for diabetes therapy ADDIN EN.CITE [31]
2012: Approval of once-weekly, extended release exenatide (Bydureon^R^) for diabetes ADDIN EN.CITE [32].
2013: Approval of liraglutide, 3 mg, for obesity therapy (Saxenda^R^) ADDIN EN.CITE [33]
2014: Approval of dulaglutide, a once weekly GLP-1 RA for diabetes [34]
2016: The LEADER trial: a cardiovascular outcome trial of liraglutide for diabetes; the cardiovascular benefits of GLP-1RAs ADDIN EN.CITE [35]
2017 : Approval of semaglutide 1 mg (Ozempic) once weekly for diabetes therapy ADDIN EN.CITE [31]
2019: Oral semaglutide (Rybelsus^R^) ADDIN EN.CITE [36]
2021: Approval of semaglutide 2.4 mg (Wegovy) for obesity therapy ADDIN EN.CITE [37]
2021-23: Introduction of tirzepatide (a weekly, dual GIP-GLP-1 agonist) for diabetes and obesity therapy ADDIN EN.CITE [38]
2023: Orforglipron, a non-peptide oral GLP-1RA (“small molecule”) ADDIN EN.CITE [39].

### GLP-1 and the endocrine pancreas

**Fig. 1:**
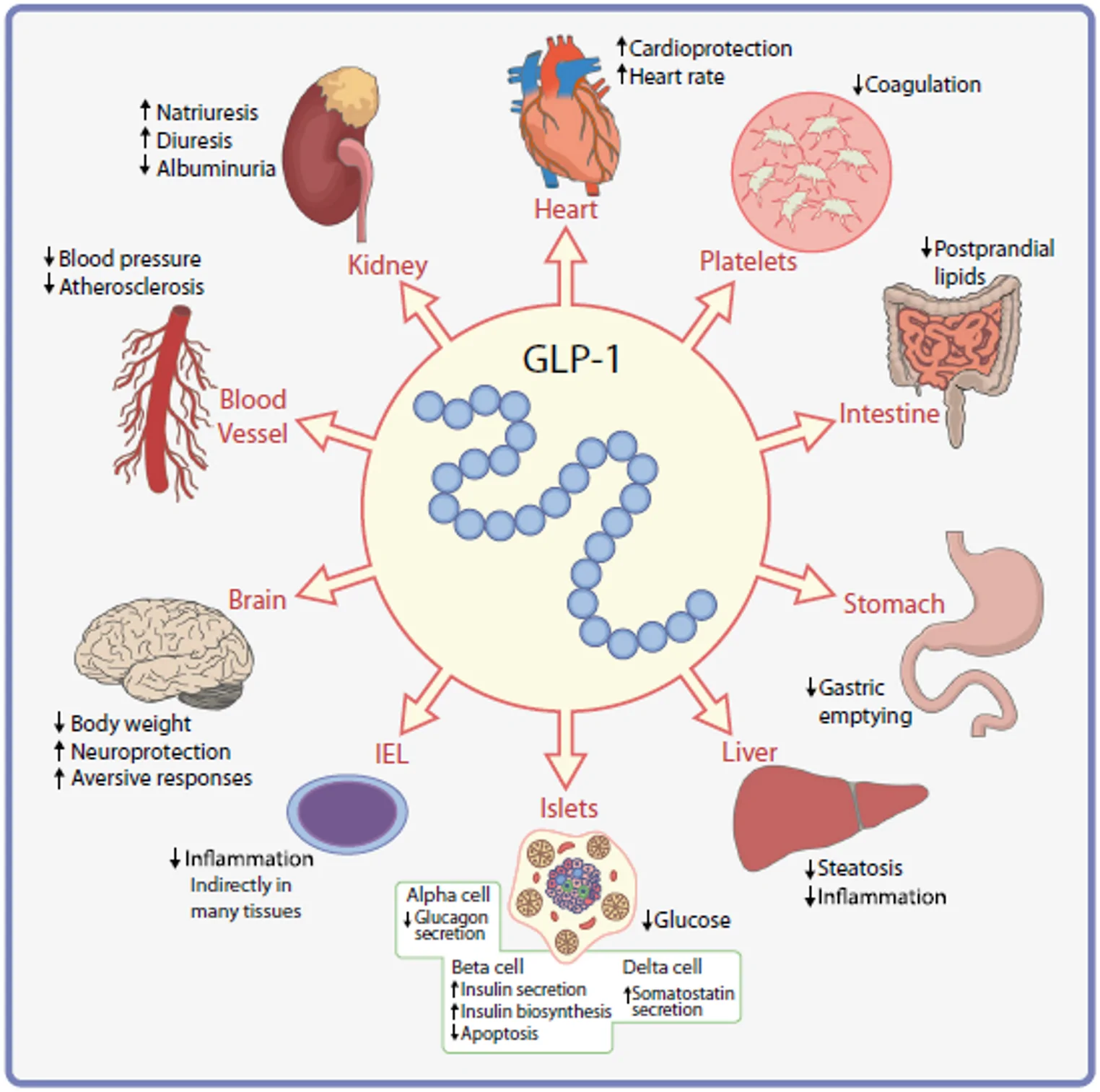
Actions of GLP-1

Physiologically, GLP-1 is usually considered an incretin hormone, one of the gut hormones responsible for the augmentation of insulin secretion after oral as opposed to intravenous administration of glucose (See Fig. 1 for an overview of GLP-1 actions). Indeed, GLP-1 was discovered as an insulinotropic peptide that could be extracted from the small intestinal mucosa [17, 18]. It fulfils the criteria for being an incretin hormone: it is secreted in response to oral administration of glucose (but to changes in arterial glucose concentrations) and acts on GLP-1 receptors expressed by the beta cells in the pancreatic islets to potentiate glucose-induced insulin secretion [19]. The GLP-1 receptor belongs to the family B of GPCRs [40] and activates G-alpha-s and thereby adenylate cyclase with formation of cAMP and downstream effects via PKA and Epac pathways leading to Ca++-dependent insulin exocytosis [41]. The effect of intestinal glucose to release GLP-1 has been worked out in some detail [42, 43] and involves glucose transport into the L-cells via SGLT-1 in cotransport with sodium ions, depolarization of the L-cell and presumably calcium entry leading to exocytosis of the contents of basolateral granules. It follows that stimulation causes release in parallel of glicentin, oxyntomodulin, GLP-1 and GlP-2 and, at least for the distal L-cells which co-express this hormone, also PYY (the upper small intestinal L-cells do not express PYY).

However, GLP-1 is more than an incretin hormone; it soon became apparent that it also potently inhibits glucagon secretion [44]. The mechanism is still debated, but evidence supports that GLP-1 acts on the somatostatin-producing delta cells and that resulting somatostatin release inhibits alpha cell secretion via somatostatin 2,3 and 5 receptors expressed by the alpha cells in most species (whereas the GLP-1 R is poorly and variably expressed in alpha cells) [45, 46].

The dual action on both insulin and glucagon secretion makes GLP-1 unique and explains that infusion of GLP-1 potently influence blood glucose by both stimulating insulin-induced glucose-disposal and by inhibiting hepatic glucose production as a combined action of stimulated insulin and inhibited glucagon secretion [47]. This can be clearly demonstrated in people where physiological infusions of GLP-1 lower fasting plasma glucose levels, while stimulating insulin secretion and inhibiting glucagon secretion, resulting in inhibition of hepatic glucose production. But because of the glucose-dependency of both the insulin stimulation and the glucagon inhibition, plasma levels of glucose do not fall very much, only about 0.5-1.0 mmol/L, despite ongoing or increasing infusions of GLP-1 [47]. The peptide therefore does not cause hypoglycemia under normal circumstances.

### Gastrointestinal effects

Other important actions of GLP-1 include its effects of the gastrointestinal organs [48]. It was observed in 1993 that it powerfully inhibits gastric emptying rate in humans, and since the gastric emptying rate is one of the most important factors regulating postprandial glucose levels [49], this effect has considerable clinical interest. It also inhibits gastric acid secretion [50] and pancreatic exocrine secretion in humans as well as in experimental animals [51]. Interestingly, the actions do not appear to be exerted directly on the GI organs (although there may also be local actions). The effects on gastric emptying and gastric and pancreatic secretion depend entirely on vagal activity and are lost after sectioning of the vagus nerves [52]. These observations gave rise the theory that GLP-1 affects vagal reflex pathways by activating sensory afferent neurons, relaying in the brain stem and the hypothalamus and inhibiting efferent vagal fibres controlling gastrointestinal motility and secretion [53]. The GI inhibitory actions are consistent with a physiological role for GLP-1 as a hormone of the “ileal brake” mechanism, the neurohormonal inhibition of gastrointestinal motility and secretion elicited by the presence of nutrients in the ileum (where the density of the L-cells is particularly high) thereby controlling entry and digestion of nutrients in the gut [54]. By simultaneously stimulating insulin secretion deposition of the nutrients already absorbed will be promoted.

### Metabolism and measurement of GLP-1

During the studies characterizing the physiological actions of GLP-1 it was noted that its half-life in the circulation is unusually short, only about 1.5–2 min and with a plasma clearance of up to 10 l/min, more than 3 times the cardiac output [25, 55]. No organ can explain elimination at this rate. The explanation is degradation catalysed by an ubiquitous enzyme, dipeptidyl peptidase-4 (DPP-4) which cleaves off the two N-terminal amino acids, and renders the resulting metabolite (GLP-1 9–36 amide) inactive [25]. This rapid elimination rate has important consequences. First, it complicates the measurement of GLP-1 in plasma [5]. Since the metabolite is inactive, it is important that the method applied for measurement of the active hormone involves the N-terminus of the molecule; the antibodies applied for radioimmunoassays or ELISAs must therefore be “terminal wrapping” to be able to measure “active GLP-1”. Antibodies that are “side viewing “ (the majority), i.e. directed towards the middle part of the sequence, will measure both the intact hormone and the metabolite (designated “total GLP-1”). The side viewing antibodies, however, will also cross-react with pancreatic products of proglucagon, in particular the MPGF and any fragments thereof. Results with such antibodies will therefore be misleading. There is an additional problem: in proglucagon GLP-1 occupies the positions 78–108, but in most species including humans, the C-terminal amino acid (Gly) functions as substrate for the PAM oxidase, leading to amidation of the preceding amino acid. This is why the full structure of human GLP-1 is designated GLP-1 [7–13, 17–19, 25, 40–58] amide. Since the amidation mainly occurs during the intestinal processing of proglucagon to GLP-1, antibodies against the *amidated* C-terminus will therefore be specific for this intestinal modification and may be used as single antibodies in radioimmunoassays for both total GLP-1 (because they are independent of the N-terminal modification and do not react with MPGF), and for measurements of intact GLP-1 as well if combined with an N-terminal wrapping antibody in a sandwich ELISA. Currently, there are no ELISAs like that available, rather the companies have used C-terminal antibodies that are not terminal wrapping, hoping to be able to include Gly-extended forms of GLP-1 as well (GLP-1 7–37), since these may be formed to an appreciably extent in for example rats and pigs. The use of single antibody assays employing terminal wrapping antibodies against the amidated C-terminus may therefore be advantageous.

### GLP-1 and DPP-4

Physiologically, the rapid N-terminal degradation of GLP-1 has important physiological consequences. It occurs already on the capillaries of the gut from where it is released (the capillaries express the DPP-4 enzyme) [56] so that very little, perhaps 10% of what is secreted eventually survives in the circulation in the intact form [57]. Plasma concentrations of active GLP-1 are therefore very low, often immeasurable, and it is very unlikely that the circulating concentrations even postprandially are sufficient to have any effects on the targets discussed above. What seems to be happening is that newly released GLP-1, which is still in the intact form, may interact with sensory afferent neurons of the vagus nerve (the GLP-1 receptor is expressed in the cell bodies of neurons of the vagal afferents in the nodose ganglion) and in the neurons of the enteric nervous system [53, 58, 59]. A similar system has been identified for other gastrointestinal hormones including cholecystokinin. The nutrient-stimulated L-cell therefore signals to these afferent neurons, which signal to the nucleus of the solitary tract in the brain stem. This is one of the key centers for gastrointestinal communication to the brain, and signals from neurons excited there may be transmitted to the dorsal vagal motor nuclei (and/or hypothalamic nuclei) as well as efferent neurons of the vagus, in this way explaining that the gastrointestinal effects of GLP-1 depend on an intact vagus function.

### GLP-1 and food intake

Another important function of the gut is regulation of food intake [60]. In 1973, CCK was demonstrated to inhibit food intake [61], and it was natural to look for similar effects of GLP-1. ICV administration of GLP-1 was demonstrated in 1996 to inhibit food intake [62], but this probably occurs via mechanisms that differ from those elicited by GLP-1 secreted from the periphery. There are numerous GLP-1 receptors in the brain [63], but they are probably targets for GLP-1 produced in and released from nerve endings of the *ppg neurons*, with cell bodies located in and around the nucleus of the solitary tract, projecting to numerous regions of the brain [64]. Gastric distension for instance, known to inhibit food intake, was demonstrated to activate those neurons [65]. Peripheral GLP-1, however, does not seem to interact directly with the ppg neurons [66]. However, in 1998 it was demonstrated that physiological *intravenous* infusions of GLP-1 inhibit appetite as well as ad libitum food intake in humans [67]. The effects could later be shown to be dose-dependent [68]. Since the effects of food intake can also be demonstrated to depend on an intact vagus nerve (also in people) [69], it is assumed that the release of intestinal GLP-1 elicits activation of ascending sensory nerves circuits that inhibit food intake. This is supported by several physiological experiments [58].

GLP-1, however, may also affect the brain via a different pathway. Indeed, during ingestion of very large meals or in individuals with accelerated gastric emptying rates (e.g. after gastric bypass) peripheral active GLP-1 concentration may rise sufficiently to influence accessible receptors. In studies mapping accessible targets of peripherally injected radiolabeled GLP-1 strong GLP-1 binding was observed in the area postrema, the subfornical organ, and the median eminence [70], all belonging to the so-called circumventricular organs, where leaky fenestrae allow circulating substances to reach brain neurons. In these regions of the brain there is also dense GLP-1 receptor expression, and during GLP-1 administration the same neurons show cFos expression as a sign of activation [71]. The activated neurons in turn transmit signals to nuclei that are important for regulation of autonomic functions as well as including food intake; target nuclei include the paraventricular and arcuate nuclei, the nucleus of the solitary tract, the parabracial nucleus, the nucleus ambiguus, the central amygdala and the bed nucleus of the stria terminalis [72]. Some of these sites are also involved in the so-called “reward mechanism “, suggesting and now experimentally supported that GLP-1 powerfully inhibits the reward mechanism as one of its important mechanisms of regulating food intake [73]. An excellent summary of the many often conflicting studies regarding the neuraleffects of both locally produced and periheralGLP-1 on appetite and food intake was provided recently by Beutler [74].

All these effects underly the formidable effects of the GLP-1Ras on body weight and diabetes. There is no effect on energy expenditure [27].

## The GLP-1 receptor agonists (GLP-1Ras)

### GLP-1 and diabetes therapy

In 1993, it was demonstrated that intravenous infusions of GLP-1 could completely normalize, in the course of 4 h, the plasma glucose concentration of individuals with longstanding severe type 2 diabetes [21]. The effect coincided with stimulation of insulin secretion and inhibition of glucagon secretion. This was a turning point in the development of the GLP-1-based diabetes therapy and clearly demonstrated the potential of the new hormone. The immediate problem was its ultrashort half-life [55]. Attempts to reproduce the effects of iv infusion by subcutaneous injections were unsuccessful and illustrated another problem – with increasing doses of the peptide to make up for the short half-life, side effects, including nausea and vomiting, began to appear [75]. Thus, also the side effects were dose dependent, preventing the use of high doses to circumvent the instability of the molecule. A demonstration of the full antidiabetic potential of GLP-1 was therefore difficult, but Zander et al. administered natural GLP-1 to people with type 2 diabetes for six weeks using continuous subcutaneous infusion [28]. This resulted in great improvements in glucose control, beta cell function, insulin sensitivity and even body weight and was virtually without side effects. And most importantly, there were no signs of tachyphylaxis to continued GLP-1 exposure. But how to leverage the promising therapeutic potential?

### DPP-4 inhibitors

The identification of the DPP-4 enzyme as the mechanism behind the short half-life led to the development of DPP-4 inhibition as a therapy for T2DM [26]. In experimental studies it was demonstrated that inhibition of the enzyme with already existing inhibitors (e.g. valine pyrrolidide) would completely protect the hormone against the N-terminal degradation, accompanied by several fold increases in glucose-induced insulin secretion [76]. Inspired by these observations, several companies developed efficient, long-acting DPP-4 inhibitors, which were demonstrated to be excellent antidiabetic agents on par with the existing oral agents (metformin, sulfonylureas) and, importantly, the DPP-4 inhibitors were almost devoid of adverse effects [77, 78]. They were particularly effective in combination with metformin, which was found to increase the secretion of GLP-1 [79]. Today, the DPP-4 inhibitors are losing their importance for mainly 3 reasons: They are, unlike the GLP-1 RAs (see below), almost without effect on body weight. There are several explanations for this. One is that, as discussed above, endogenous GLP-1 probably mainly acts via activation of sensory afferent neural pathways to the brain- and this activation is local, occurring in the mucosa of the gut, and before the hormone is degraded by the enzyme, which occurs when it is taken up by the capillaries that drain the mucosa [56]. The means that the presence or absence of DPP-4 activity doesn’t matter a lot for the activation of the satiation mechanism. It is also possible that increases in active GLP-1 levels brought about by DPP-4 inhibitors are insufficient for inhibition of appetite. Finally, while circulating GLP-1 is protected against degradation by DPP-4, the other important hormone PYY, *needs* DPP-4 activity to be converted to the truncated form PYY 3–36, which is the form acting on hypothalamic Y2 receptors to inhibit appetite and food intake [80]. Thus, the PYY mediated satiation mechanism is lost after DPP-4 inhibition. Importantly and unlike treatment with the GLP-1 receptor agonists, DPP-4 inhibitor treatment is *not* associated with a decreased cardiovascular risk, as documented in very large trials, including the TECOS trials with the most important inhibitor, sitagliptin. These trials largely documented *absence* of increased risk, but also no superiority compared to placebo [81]. There may be microvascular and renal improvements during therapy, but despite this there were no effects on MACE, major cardiovascular adverse events. The DPP-4 inhibitors therefore, although rather similar in terms of antidiabetic control, cannot be recommended compared to the SGLT2 inhibitors that appeared on the market after the introduction of the DPP-4 inhibitors. Treatment with the SGLT2 inhibitors not only associate with lowering of glucose *and* body weight, but also have beneficial cardiovascular effects [82]. However, for people with problems with the existing oral therapies, the DPP-4 inhibitors remain an attractive possibility.

### GLP-1 analogs, the GLP-1 receptor agonists

An alternative approach to leverage the therapeutic potential of GLP-1 was to stabilize the peptide against DPP-4. This turned out to be relatively easy, since all it took was substitution of residue no 2 with another amino acid that would not impair receptor activity. Several amino acids were demonstrated to qualify for this (Ser, Val, Gly and Aib (alpha-iso-butyric acid)) [83]. However, an additional problem remained: renal clearance. GLP-1 was demonstrated to be efficiently cleared by the kidneys [84], resulting in a half-life for DPP-4 stabilized analogs of 5 min [83], clearly insufficient for clinical application.

Exenatide, the synthetic form of the salivary gland peptide, exendin 4, from the Gila monster, turned out to provide a solution. Exenatide shares about 50% sequence similarity with GLP-1 and is a full and equipotent agonist for the GLP-1 receptor [29]. It is resistant to the actions of DPP-4 and is cleared in the kidneys by glomerular filtration only [85]. This means that it has an intravenous half-life of about 30 min in humans, and the plasma half-life after subcutaneous injections is about 2 h. When injected before a meal the postprandial glucose excursions are completely flattened out and this applies to healthy individuals as well as patients with type 2 diabetes [29]. Upon chronic administration a weight loss of a couple of kg is also seen. The particular importance of the early introduction of exenatide (approved in 2005 under the trade name Byetta) was that it provided proof for the concept that a GLP-1 receptor agonist could be used in practical clinical treatment of type 2 diabetes. Eventually, exenatide was developed into a slow-release formulation that could be given once a weak (Bydureon) with better performance [86]. The clinical effect of Byetta was almost entirely due to the inhibition of gastric emptying, and therefore the effect was strong on postprandial glucose excursions but not so clear on fasting glucose concentrations. The weekly formulation obviously performed better in this respect.

However, it was the introduction of acylated GLP-1 by NovoNordisk that finally solved the problem of the renal elimination. By attaching a fatty acid chain to the GLP-1 molecule, the analog would bind to albumin in the circulation and avoid both DPP-4 activity and renal elimination. Indeed, the acylated GLP-1, liraglutide (Victoza) has a half-life of 12 h and provides continuous plasma exposure with once-daily injections [31, 87]. The clinical results with liraglutide, approved in 2009-10, were good (the LEAD trials) [87], also compared with the other available GLP-1RAs and weight losses around 4 kg were observed. However, more importantly, a cardiovascular outcome trial conducted with liraglutide showed that not only did it not increase cardiovascular risk (the outcome trials were demanded by the FDA [88] because another relatively new antidiabetic agent, rosiglitazone, was suspected to increase cardiovascular risk), but – like the SGLT2 inhibitors - it was actually superior to placebo and decreased risk by 13% in a trial (the LEADER trial )of 9000 individuals lasting for up to 5 years [35]. The problem with the side effects of nausea and vomiting was still evident, but was mainly occurring early during treatment and could be significantly reduced by slow up titration of dosing [89].

Given that the effects on food intake and therefore presumably also weight loss would be dose dependent [68], a new dose response study with adequately slow up titration showed that increasing the dose from the usual 1.2–1.8 mg up to 3 mg would considerably increase the weight loss over one year, now amounting to 7–8% [90], and this inspired the company NovoNordisk to conduct a phase 3 trial in people with overweight /obesity but without diabetes, the so-called SCALE trials [33]. The findings of the phase 2 trial were confirmed and 3 mg liraglutide was now, as the first GLP-1 receptor agonist, approved for obesity treatment, under the trade name Saxenda.

### Weekly GLP-1RAs

The weekly administration of Bydureon (weekly exenatide) was of course a significant advantage (although there were some problems with subcutaneous nodules at the injection sites), and one of the companies behind (Eli Lilly) also managed to solve the instability problem of GLP-1 by attaching GLP-1 to a another large molecule (an Fc fragment ), dulaglutide, preventing glomerular filtration and allowing weakly dosing [34].

At Novonordisk, a second generation GLP-1 receptor agonist, semaglutide, was now developed. It was stabilized further against DPP-4 and bound more strongly to albumin resulting in a half-life of 1 week [31]. The registration trials (the SUSTAIN trials) were very successful, and semaglutide was now clearly the leading antidiabetic compound in the field [91]. The weight loss was on par with that of Saxenda, and the reduction of cardiovascular risk in a pre-registration cardiovascular outcome trial (SUSTAIN 6) was 26% [91]. A slow up titration schedule ensured tolerable side effects. Once again, however, it was decided to run a dose-response study in obesity without diabetes, using daily dosing to ensure very small increments in dose throughout the study [92]. Here, the highest dose, 0.4 mg/day caused a weight loss over one year of 14–15%. This was a major breakthrough, and a phase 3 program, the STEP trials, was conducted with semaglutide at 2.4 mg once weekly (Wegovy). A very slow up titration schedule was applied (20 weeks) ensuring tolerable side effects. The step trials were very successful showing weight losses of up to 18% over 68 weeks [93]. These results changed the attitude to treatment of overweight with GLP-1RAs, a therapy that immediately became extremely popular and highly sought-after.

### The development of co-agonists

The competing company, Eli Lilly, also developed a second generation GLP-1RA, but chose to follow up on previous reports on experimental compounds that would combine the actions of more than one hormone in a monomolecular form. The phase three program of the agonist, tirzepatide, combining GIP and GLP-1 activity (GIP is the other incretin hormone) was extremely successful [94] and demonstrated HbA1c reductions below 5.7% (i.e. normal values! ) in more than 50% of patients treated with 15 mg dose [95]. This is the best result ever observed and completely changes our view on diabetes treatment. Also regarding weight loss, tirzepatide appeared superior. In the SURMOUNT trials of nondiabetic obesity, weight losses over 88 weeks of almost 26% were observed [96]. Importantly, in a 3 year extension of the SURMOUNT 1 trial, the risk of incident type 2 diabetes, which is high in this group of patients, was almost eliminated by tirzepatide treatment (by 94%) [97] .

An even greater weight loss may be obtained with the triple agonist, retatrutide, agonizing boh the GLP-1, the GIP and the glucagon-receptor. Indeed, in the phase and 2 3 trials weight losses approaching 30% have been observed. The inclusion of glucagon agonism apparently provides additional weight losing properties, and perhaps most importantly counteracts steatosis and inflammation of the liver (MASLD), which often accompany type 2 diabetes and obesity [98]. Efficacy in diabetes treatment is apparently not compromised.

Because of the effectiveness in the treatment of hepatic steatosis. a number of GLP-1 glucagon coagonists have been developed [9]. Common for all, is their effectiveness in hepatic steatosis but their efficacy in diabetes and obesity treatment varies. One, mazdutide, has been approved in China [99].

Semaglutide was also subjected to a cardiovascular outcome trial in the obese population (already with some cardiovascular impairment), the SELECT trial of 17.600 people with obesity treated with 2.4 mg for nearly 4 years [100]. The risk of MACE was reduced by 20%. This is of course an extremely important result because it shows that the increased cardiovascular risk in a population of overweight obese individuals, which may correspond to up to a quarter of the adult population of many countries, can be alleviated by therapy with a GLP-1 Receptor agonist. There are several other remarkable benefits of the therapy with the potent GLP-1RAs: obesity associated cancer risk may also be significantly reduced by the therapy – thus the risk of developing 10 of 13 obesity associated cancer types was significantly reduced during therapy with GLPRAs [101]. Also, renal function may be significantly improved as demonstrated in the FLOW trial of 3.500 individuals with diabetic kidney disease, where the fall in glomerular filtration rate observed in this population could be significantly delayed [102]. Another exciting consequence of therapy is improved cognitive function [103] and significant reductions in incidence of dementia during treatment [104]. Clinical trials investigating possible beneficial effects on the risk of developing of Alzheimer’s disease (the EVOKE trials) however, did not show effects on progression in early-stage Alzheimer’s disease [105], whereas observational, real-World studies had indicated highly significant effect on dementia development in general [106, 107].

Presumably as a result of the interactions of the GLP-1RAs with reward mechanisms in the brain [73], the agonists have also shown beneficial results in studies of abuse of both opioids and alcohol [108].

Probably the best underlying explanation for all these beneficial outcomes may be found in the following three important factors: the weight loss, which in itself has marked effects on cardiovascular and metabolic health; a surprising and pronounced anti-inflammatory effect, which shows up in the improving concentrations of circulating inflammatory markers, with particular and marked dose-dependent reductions in high sensitivity CRP levels [109]; in finally, an effect on the vascular system [110]. Several experimental as well as clinical studies have suggested beneficial actions on the blood vessels including preventive effects on experimental atherosclerosis [41]. Indeed, semaglutide was recently demonstrated to show highly beneficial effects on peripheral artery disease in the clinical STRIDE study [111]. These three main factors are all diffuse with respect to their targets which apply to the whole body and therefore may provide an explanation for the beneficial effects of GLP-1Ras on numerous different organs many of which do not even excess GLP-1 receptors: the lungs, the heart, the liver, the kidneys and the central nervous system (where most of the receptors are both available for peripheral GLP-1RAs. It is of interest to compare the treatments with the GLP-1RAs with bariatric surgery where at least a very important part of the health and weight promoting effect involves an exaggerated release of gut hormones (GLP-1 and PYY) [80].

### Orally active agonists

The need for injection of the peptide-based GLP-1RAs is by many considered a major problem, but orally available GLP-1 RAs are now available. NovoNordisk co-formulated semaglutide with SNAC, an absorption enhancer allowing rapid absorption of the active ingredient already in the stomach [36]. The bioavailability is low, but once absorbed, the compound behaves precisely as injected semaglutide with similar effects (and side effects) for the same plasma exposure [112]. Oral semaglutide has been approved for both diabetes and obesity therapy, and the effects at a daily dose of 25 mg are similar to those obtained with 2.4 mg s.c [113]. At Eli Lilly, a non-peptide small molecule agonist of the GLP-1, orforglipron, has been developed. It shares effects (and side effects) with the injectables, and has been approved for both diabetes and obesity therapy [39]. It may represent an important advance, since the small molecules are probably easier and cheaper to produce in large quantities.

### Negative consequences of GLP-1-based obesity therapy

The entire class of GLP-1RAs (including co-agonists) is associated with so-called gastrointestinal side effects [114], nausea, vomiting and altered defecation habits, but in most cases, these can be avoided by slow increases in dosing [115]. Indeed, the titration period may last for half a year or more. The important message is patience, and not everybody needs to reach the maximum dose. Recent results with the REDEFINE trial of the combination of semaglutide and cagrilintide (Cagrisema) suggest that lower doses not associated with nausea do not necessarily produce lesser weight losses [116]. However, 5–10% may stop treatment because of the side effects, which should therefore be avoided.

The weight loss induced by the GLP-1RAs is accompanied by losses of lean body mass -there is no indication that the compounds have weight-loss independent effects on lean body mass (except maybe for GLP-1 and glucagon co-agonists) [117]. The loss of lean body mass can be avoided/slowed down by nutritional precautions such as ensuring adequate protein intake despite the lower overall food intake. An effective mitigation is obtained by increased physical activity, which has additional metabolic and cardiovascular benefits, and is therefore extremely important [118, 119]. Obviously, adequate micronutrient intake should also be ensured.

A number of rare side effects are being investigated for association with GLP-1RA treatment. A slightly increased incidence of diseases of the biliary system is probably real, but other suggested side effects are typically also associated with weight loss and different to ascribe to the GLP-1RA therapy [114].

The most serious problem, however, is the weight gain that generally follows GLP-1RA termination after weight loss [120]. The weight gain will predominantly be composed of fat, which means that the individual may end up with a poorer fat-mass/lean-mass ratio than before treatment which can have serious health consequences. It is therefore necessary for the prescriber as well as the patient to make a long-term plan for the therapy from the beginning. If new effective diet and physical activity habits have been established, it may be possible to maintain the weight loss and thus the health status. In other cases, it may be possible to maintain the weight loss by lowering the dose, supported by the experience with “microdosing” ( despite being introduced to reduce costs) [121]. These problems are not trivial in a situation where there is a shortage of drug and prices are prohibitive, but recent developments with production of generic GLP-1 RAs by numerous companies in several countries would seem to limit these problems.

### Are the GLP-1RAs longevity drugs ?

The significant decreases in all-cause mortality observed in the large cohorts of the cardiovascular outcome trials would indeed suggest that survival may be effected by GLp-1 therapy. It is tempting to compare with the weight loss obtained by bariatric surgery, which is also associated with pronounced improvements in overall health and reduced complications to obesity and incidence of diabetes and importantly increases lifetime-expectancy, by 9 years for those who had diabetes at the time of operation and by 5 years for those without [122]. In view of the similarity of mechanisms, it is tempting to speculate that a similar improvement of lifespan and health will be the consequence of GLP-1RA treatment. Appreciating their importance, the GLP1RAs have now been included in the WHO list of “essential medicines” and are being recommended for treatment of obesity in combination with life style changes [123].
